# Integrated metagenomic and metabolomic profiling of spontaneous preterm birth in Chinese women

**DOI:** 10.3389/fmicb.2026.1729476

**Published:** 2026-03-18

**Authors:** Heng Xue, Mengjun Zhang, Yao Tang, Wu Huang, Xingliang Yu, Jun Zhang, Mian Pan, Zhaodong Liu

**Affiliations:** 1 Department of Laboratory Medicine, Fujian Maternity and Child Health Hospital, College of Clinical Medicine for Obstetrics & Gynecology and Pediatrics, Fujian Medical University, Fuzhou, Fujian, China; 2 Department of Obstetrics and Gynecology, Fujian Maternity and Child Health Hospital, College of Clinical Medicine for Obstetrics & Gynecology and Pediatrics, Fujian Medical University, Fuzhou, Fujian, China; 3 Department of Laboratory Medicine, School of Medical Technology and Engineering, Fujian Medical University, Fuzhou, Fujian, China; 4 Key Laboratory of Clinical Laboratory Technology for Precision Medicine (Fujian Medical University), Fujian Province University, Fuzhou, Fujian, China

**Keywords:** biomarkers, early prediction method, metabolomics, metagenomics, spontaneous preterm birth, vaginal microbiota

## Abstract

**Background:**

Spontaneous preterm birth (sPTB) remains a major cause of neonatal morbidity and mortality. We used integrated metagenomics and untargeted metabolomics to identify vaginal microbial and host metabolic signatures associated with sPTB in Chinese women.

**Methods:**

Vaginal swabs (sPTB, *n* = 37; term, *n* = 62) and available maternal plasma were profiled by shotgun metagenomic sequencing and UHPLC–HRMS metabolomics. Group differences in microbial diversity/taxa and metabolite features were evaluated, followed by pathway enrichment and microbiome–metabolome correlation analyses.

**Results:**

Compared with term controls, sPTB was characterized by reduced Lactobacillus dominance, higher vaginal microbial alpha diversity (*p* < 0.05), and distinct community structure (PERMANOVA *p* < 0.001). Metabolomic profiles of plasma and vaginal fluid differentiated sPTB from term pregnancy and highlighted decreased pantothenic acid and increased 4-pyridoxic acid, together with lipid and amino-acid perturbations. Pantothenic acid showed good discrimination (AUC = 0.82), and a multi-metabolite model improved classification (AUROC = 0.9544). KEGG analysis implicated vitamin B6 metabolism, pantothenate/CoA biosynthesis, and glycerophospholipid metabolism. Microbiome–metabolome integration dentified exploratory an sPTB-associated pattern in which Lactobacillus (e.g., *L. crispatus*) was positively correlated with pantothenic acid, while dysbiosis−/pathogen-associated taxa (including *C. trachomatis*) correlated with 4-pyridoxic acid.

**Conclusion:**

sPTB in this Chinese cohort is associated with concurrent vaginal dysbiosis and systemic/local metabolic disturbances, supporting integrated microbiome–metabolite markers for risk stratification and potential preventive targets.

## 1 Introduction

Preterm birth, i.e., delivery at <37 weeks’ gestation, is a worldwide health issue, complicating ~10% of pregnancies worldwide (Blencowe et al., 2012; Feehily et al., 2020). It is the leading cause of neonatal mortality and a major cause of under-5 childhood mortality (Ng et al., 2021). Spontaneous preterm birth (sPTB), caused by spontaneous labor or preterm rupture of the membranes, accounts for the majority. The etiopathogenesis of sPTB is multifactorial and complex and involves uteroplacental ischemia, maternal-fetal stress, immune-inflammation, and infection (Schuster et al., 2024). Responsible for a direct proportion of as much as 40% ~ 50% of sPTB are infection and inflammation ascending through the reproductive tract (Cavanagh et al., 2025). Vaginal environment is pivotal to pregnancy outcomes. A healthy pregnancy is typically typified by a low-diversity, *Lactobacillus*-prevailing vaginal microbiota that is lactic acid-producing, of low pH, and pathogen-protective (Feehily et al., 2020; Ng et al., 2021; Ravel et al., 2011). In contrast, vaginal dysbiosis in terms of *Lactobacillus* depletion and anaerobic overgrowth (e.g., in bacterial vaginosis, BV) has been linked to adverse outcomes like sPTB (Feehily et al., 2020; Ng et al., 2021). A *Lactobacillus*-free vaginal community with high diversity (typically referred to as community state type IV) is a risk predictor of sPTB (Fettweis et al., 2019; Freitas et al., 2018; Ng et al., 2021; Tamarelle et al., 2018). BV women, for example, are approximately twice as likely to experience preterm delivery (Ng et al., 2021). Some of the bacteria being linked to dysbiosis, e.g., *Gardnerella*, *Atopobium*, *Prevotella*, Sneathia and others, have been repeatedly shown to be involved in PTB across different populations (Feehily et al., 2020; Fettweis et al., 2019). In contrast, presence of *Lactobacillus crispatus* is uniformly linked with term delivery and reduced risk of PTB (Feehily et al., 2020; Fettweis et al., 2019).

Even with extensive investigation, researchers have yet to identify a microbe or mechanism that completely accounts for sPTB. Existing literature shows population-specific differences in relationships between PTB and vaginal microbiota (Kumar et al., 2021; Ng et al., 2021). Interestingly, a large cohort study conducted in China found that the composition of mid-pregnancy vaginal microbiota was not a significant predictor of PTB in a low-risk cohort (Ng et al., 2021), hypothesizing that underlying differences in either host immunity or microbiota might be responsible for generally lower PTB rates in Asian women. This cohort did, nevertheless, have low PTB incidence overall and received interventions (e.g., BV treatment) that could have obscured associations (Schuster et al., 2024). Conversely, research among other populations has reported notable associations between vaginal dysbiosis and sPTB risk (Feehily et al., 2020; Fettweis et al., 2019). These discrepancies highlight the necessity of investigating the vaginal microbiome in diverse ethnic and clinical contexts. In particular, integrated data on the vaginal microbiome and metabolic alterations in Chinese women with sPTB remain scarce.

Beyond the microbiome, maternal metabolic health is increasingly becoming a piece of the preterm birth puzzle. Pregnancy entails profound metabolic changes; maternal-fetal metabolic imbalance can predispose to complications like PTB (Cavanagh et al., 2025). Recent metabolomics have also found biochemical markers and pathways associated with PTB, such as lipid metabolism, amino acid turnover, and oxidative stress markers (Amabebe et al., 2016; Mercer et al., 2023). B vitamins (pantothenic acid [B5] and pyridoxine [B6]) are cofactors in numerous metabolic reactions and are associated with pregnancy outcomes (Carter et al., 2019). For instance, reduced levels of plasma pyridoxal-5′-phosphate (vitamin B6) have been associated with an enhanced risk of PTB (Barinov et al., 2022). The inflammation-regulated tryptophan metabolism via the kynurenine pathway is also a pathway involved in PTB and infection; an elevated vaginal kynurenine/tryptophan ratio has been correlated with *C. trachomatis* infection and unfavorable outcomes (Chen and Guillemin, 2009; He et al., 2020; Ziklo et al., 2018). Microbiome–metabolome interactions are therefore most likely to be central: vaginal microbes have the potential to dictate local metabolite pools, while metabolites dictate microbial growth and host immunity (Cavanagh et al., 2025; Ziklo et al., 2018). An integrated microbiome-metabolome approach could therefore shed light on pathogenic processes in sPTB that cannot be elucidated from either data type alone (Ansari et al., 2024; Cavanagh et al., 2025; Flaviani et al., 2021).

We applied here a multi-omics strategy combining metagenomics and metabolomics to study sPTB in a Chinese cohort of pregnant women. By describing the vaginal microbial community and maternal plasma and vaginal secretion metabolic profile, we aimed to: (i) establish microbiome changes associated with sPTB (e.g., decrease in *Lactobacillus* and increase in putative pathogens), (ii) characterize significant metabolite changes in sPTB (such as changes in vitamin- and amino acid-related metabolites), and (iii) examine microbiome-metabolite associations to generate mechanistic hypotheses regarding how vaginal dysbiosis and host metabolism may jointly contribute to preterm birth. We hypothesized that sPTB in this population would be characterized by a dysbiotic, *Lactobacillus*-depleted vaginal microbiome alongside disruptions in host metabolic pathways (particularly involving B vitamins and lipid metabolism), and that these processes would be inter-related. To our knowledge, this is among the first studies in Chinese women to integrate shotgun metagenomic sequencing with broad-spectrum metabolomics to characterize microbial and metabolic signatures associated with sPTB. Elucidating these interactions may reveal novel biomarkers for early prediction and suggest preventive or therapeutic targets to reduce the burden of preterm birth.

## 2 Methods

### 2.1 Study design and participants

This case–control study was conducted at Fujian Maternity and Child Health Hospital (Fuzhou, China). Pregnant women were prospectively enrolled during mid-gestation after obtaining written informed consent. The study protocol was approved by the Ethics Committee of Fujian Maternity and Child Health Hospital (Approval number: 2024KY184-03).

Cases were defined as women who subsequently (after the study sampling) experienced spontaneous preterm birth (sPTB), i.e., spontaneous onset of labor or premature rupture of membranes leading to delivery before 37^+0 weeks’ gestation. Controls were women with term delivery (≥39^+0 weeks) and no obstetric complications. Exclusion criteria for both groups included multiple gestation, medically indicated preterm delivery, known cervical insufficiency, placenta previa, preeclampsia, or any prophylactic interventions for preterm prevention (e.g., cervical cerclage or progesterone). Women with acute clinical infections at the time of sampling (such as symptomatic vaginitis or urinary tract infection) were excluded to minimize confounding. Symptomatic vaginitis was defined as the presence of patient-reported genital symptoms (e.g., abnormal vaginal discharge, malodor, vulvovaginal pruritus, burning/irritation, or dysuria) and/or clinician-noted signs consistent with vaginitis on pelvic examination at the sampling visit; women meeting any of these criteria were not enrolled. Therefore, detection of organisms such as *Chlamydia trachomatis* in metagenomic data reflects asymptomatic carriage/colonization at sampling rather than clinically symptomatic infection.

In total, 37 sPTB cases and 62 term controls were enrolled. All 99 participants contributed vaginal swab samples for metagenomic sequencing. For metabolomic analyses, a subset of samples was available: vaginal fluid was obtained from all participants (37 sPTB and 62 term), whereas plasma samples were available from 21 sPTB cases and 35 term controls. Plasma metabolomics was therefore performed on the subset with sufficient remaining volume and acceptable pre-analytical quality for UHPLC-HRMS (e.g., adequate volume after prior processing/storage and absence of gross hemolysis). Because plasma was not available for all enrolled participants, we evaluated potential selection bias by comparing key baseline characteristics and pregnancy outcome distribution between participants with versus without available plasma; these comparisons are summarized in the Supplementary materials. The precise numbers of samples used for each analysis are indicated in the Results section and in figure/table legends.

### 2.2 Sample collection

For each participant, two types of samples were obtained once at enrollment during mid-gestation (mid-trimester antenatal visit), prior to the onset of labor and prior to any rupture of membranes: a vaginal swab and a peripheral blood sample. Vaginal swabs were collected by an obstetrician using two sterile cotton swabs simultaneously inserted into the posterior fornix and then rotated to achieve symmetrical sampling. Swabs were placed into DNA stabilization buffer on ice immediately. A 5 mL maternal venous blood sample was drawn into EDTA tubes, and plasma was separated by centrifugation (1,500 × g, 10 min) within 2 h. An aliquot of the vaginal swab supernatant (after pelleting for metagenomic analysis) was also retained as “vaginal fluid” for metabolomic profiling. All specimens were stored at −80 °C until analysis.

### 2.3 Metagenomic DNA extraction and sequencing

DNA was recovered from vaginal swabs with the Qiagen PowerSoil kit with the modification of mechanical lysis by bead beating to improve the yield of gram-positive bacteria. DNA quantity was quantified on a Qubit fluorometer and purity on the A260/280 ratio. Shotgun metagenomic libraries were subsequently prepared with the Illumina Nextera DNA Flex kit and sequenced on the Illumina NovaSeq 6,000 platform, generating paired-end 150 bp reads. Negative extraction controls and library no-template controls were also run at this point to identify any possible contamination.

### 2.4 Metagenomic bioinformatic analysis

Raw sequencing reads were adapter-trimmed and low-quality base-trimmed with Trimmomatic, and host-derived sequences were removed by mapping against the human reference genome (hg38) with Bowtie2. Taxonomic classification of the high-quality reads left behind was done using Kraken2 against a large microbial database containing bacterial, viral, and fungal genomes. Species-level relative abundances were estimated using Bracken. As viral and fungal sequences were detected at very low abundance, downstream analyses focused on bacterial taxa. Alpha diversity (Shannon index) was calculated from genus-level profiles to reduce assembly bias, and group differences were assessed using the Wilcoxon rank-sum test. Beta diversity was evaluated with Bray–Curtis dissimilarities and visualized by principal coordinates analysis (PCoA); the statistical significance of group clustering was tested with PERMANOVA (999 permutations). Differentially abundant taxa between sPTB and term groups were identified using both LEfSe (Linear Discriminant Analysis Effect Size) and DESeq2, applying a false discovery rate (FDR) threshold of *q* < 0.1. Only taxa consistently identified as differential across methods were reported. In addition, metagenomic data were specifically screened for established vaginal pathogens to compare their prevalence between groups. For each organism, we report detection rate as the proportion of samples with metagenomic detection (*n*, %) in sPTB versus term controls, including *Chlamydia trachomatis*, *Gardnerella vaginalis*, and Ureaplasma spp., to complement relative-abundance analyses.

### 2.5 Untargeted metabolomic profiling

Untargeted metabolomic profiling of the maternal plasma and vaginal fluid samples was conducted using ultra-high-performance liquid chromatography in combination with high-resolution mass spectrometry (UHPLC-HRMS). Methanol precipitated the plasma proteins, while vaginal fluid samples were diluted and filtered before being analyzed. Both reverse-phase C18 chromatography (for lipophilic metabolites) and hydrophilic interaction chromatography (HILIC, for polar metabolites) were employed, both in positive and negative electrospray ionization modes, to ensure a broad range of metabolome coverage. Mass spectrometry data were acquired on a Thermo Orbitrap instrument at a resolution of 120,000, scanning a mass-to-charge (m/z) range of 50–1,000.

### 2.6 Metabolite data processing

Raw spectra were calibrated and converted to mzML format. Feature detection, alignment, and deconvolution were performed using XCMS. Detected features (retention time–m/z pairs) were annotated by matching against reference metabolite libraries (HMDB, KEGG, and an in-house Chinese metabolome database) based on accurate mass (±5 ppm) and retention index. Where available, MS/MS fragmentation spectra were compared to authentic standards for confirmation of metabolite identities. Data were normalized using quantile normalization, and batch effects were corrected with QC sample-based robust LOESS.

### 2.7 Statistical analysis of metabolomics

Multivariate analyses were performed to evaluate global metabolic differences between the sPTB and term groups. Principal component analysis (PCA) was used for dimensionality reduction and visualization of overall clustering patterns. Supervised partial least squares–discriminant analysis (PLS-DA) models were subsequently constructed to maximize group separation, and model robustness was evaluated by 7-fold cross-validation with permutation testing (100 iterations) to reduce the risk of overfitting. Given the exploratory nature of untargeted metabolomics and the limited number of plasma samples (21 sPTB vs. 35 term), we additionally considered statistical power: for a two-group comparison at *α* = 0.05, this sample size provides ~80% power to detect only relatively large standardized differences (approximately Cohen’s d ≈ 0.8), and power is expected to be lower under multiple-testing control. Accordingly, findings from plasma metabolomics should be interpreted as hypothesis-generating and prioritization-oriented rather than definitive.

Key discriminating metabolites were identified based on PLS-DA loadings and further evaluated by univariate analyses using Student’s *t* test or Wilcoxon rank-sum test, as appropriate, with false discovery rate (FDR) correction. Metabolites with a variable importance in projection (VIP) score > 1 and an FDR-adjusted *q* < 0.05 were considered significantly different between groups.

To evaluate predictive capacity, receiver operating characteristic (ROC) curve analyses were performed for individual candidate biomarker based on metabolite abundance values. The area under the curve (AUC) and corresponding 95% confidence intervals was estimated using bootstrap resampling (1,000 iterations). For multi-metabolite prediction, the dataset was first split using stratified sampling into a training set (80%) and a held-out test set (20%); the test set was not used for any feature selection or model tuning. Within the training set, feature selection and hyperparameter tuning were performed using nested cross-validation (inner loop for feature selection/hyperparameter tuning; outer loop for performance estimation). A random forest classifier (500 trees) was trained within each training fold, and feature importance was ranked according to the mean decrease in the Gini index; the number of metabolites retained was determined within the inner loop only. The final model was refit on the full training set using the selected metabolites and then evaluated once on the untouched 20% test set, with AUROC and 95% CI computed by bootstrapping test-set predictions (*n* = 1,000). The top approximately 10 metabolites identified in the training procedure were subsequently incorporated into a logistic regression model, which was validated using the same train/test split and nested cross-validation scheme, and reported with AUROC and 95% CI.

### 2.8 Pathway enrichment analysis

Metabolite set enrichment analysis was performed using the Kyoto Encyclopedia of Genes and Genomes (KEGG) pathway database. Differential metabolites (q < 0.05) were mapped to KEGG identifiers, and pathway enrichment was assessed with a hypergeometric test using all detected metabolites as the background. False discovery rate (FDR) correction was applied, and pathways with an adjusted *p* < 0.05 were considered significantly enriched. Particular attention was given to pathways related to microbiota–host interactions, including vitamin metabolism, amino acid metabolism, and lipid signaling.

### 2.9 Microbiome-metabolome integration

For integrating microbiome and metabolome data, Spearman correlation analysis among relative abundances of key bacterial taxa and significantly changed levels of differential metabolites from all samples was carried out. Correlation was evaluated for the significantly altered taxa and metabolites of sPTB (e.g., *Lactobacillus* spp., *Chlamydia trachomatis*, *Streptococcus pneumoniae* vs. pantothenic acid, 4-pyridoxic acid). Correlation matrices were visualized as clustered heatmaps. Given the number of taxa–metabolite pairs tested, *p* values were adjusted for multiple comparisons using the Benjamini–Hochberg false discovery rate (FDR), and associations with q < 0.05 were considered statistically significant. These correlation analyses were intended to be exploratory and hypothesis-generating.

In addition, sparse canonical correlation analysis (sCCA) was applied to identify latent associations between microbiome composition (family-level abundances) and metabolomic profiles. This approach enabled the detection of co-varying microbe–metabolite modules characteristic of sPTB.

## 3 Results

### 3.1 Participant characteristics

A total of 99 pregnant women were enrolled in the study, comprising 37 cases of spontaneous preterm birth (sPTB) and 62 term deliveries. All participants provided vaginal swab samples for metagenomic sequencing. For untargeted metabolomics, vaginal fluid was available from all participants (37 sPTB and 62 term), whereas plasma samples were available from a subset only (21 sPTB and 35 term) because not all blood draws yielded plasma of sufficient volume and quality for untargeted metabolomics, yielding a total of 56 plasma samples. To assess whether missing plasma could introduce selection bias, baseline maternal characteristics and sampling-related variables were compared between participants with versus without available plasma; the results are provided in the Supplementary materials and are discussed as a limitation. The exact sample sizes used in each analysis are indicated in the Results and in the corresponding figure and table legends.

The clinical characteristics of the study participants are summarized in Table 1. The median gestational age at delivery was 34 + 5 weeks (range 18 + 5–36 + 6) in the sPTB group and 39 + 5 weeks (range 37 + 0–41 + 2) in the term group. There were no significant differences in maternal age, pre-pregnancy body mass index (BMI), gravidity, parity, history of abortion, vaginal pH, or gestational age at sampling between groups (all *p* > 0.1). No participants were positive for HIV infection; three women were hepatitis B surface antigen–positive. All women tested negative for syphilis serology. Antibiotic exposure during pregnancy was minimal (restricted to prophylaxis for group B *Streptococcus* or treatment of urinary tract infection), and no participants had received antibiotics within 4 weeks prior to sampling. Progesterone prophylaxis was not used in any participants, consistent with local guidelines for low-risk women. Detection rates of the five pre-specified key pathogens were generally similar between the sPTB and term groups, with no statistically significant differences observed. Overall, the two groups were comparable in baseline characteristics aside from differences in pregnancy outcome.

**Table 1:** Baseline characteristics of the study participants.

	Preterm birth (*N* = 37)	Term birth (*N* = 62)	*p* value
Age (y)	29.00 (26.00–32.50)	28.00 (26.00–32.00)	0.691
Body mass index	21.22 (20.19–24.08)	20.70 (19.12–22.76)	0.086
Obstetric history			
Gravidity	2.00 (1.00–3.00)	2.00 (1.00–2.00)	0.117
Parity	2.00 (1.00–2.00)	1.00 (1.00–2.00)	0.115
History of abortion (spontaneous or induced)			0.612
Yes (*n*, %)	16 (43.24%)	24 (38.10%)	
No (*n*, %)	21 (56.76%)	39 (61.90%)	
Collection time (gestational weeks)	23.71 (17.86–25.79)	24.14 (21.00–25.57)	0.537
Vaginal PH	4.40 (4.00–4.40)	4.20 (4.00–4.60)	0.173
Pathogen detection in metagenomic data			
*Chlamydia trachomatis*, detected (*n*, %)	3 (8.1)	4 (6.5)	0.720
*Gardnerella vaginalis*, detected (*n*, %)	24 (64.9)	34 (54.8)	0.340
*Ureaplasma* spp., detected (*n*, %)	20 (54.1)	32 (51.6)	0.830
*Ureaplasma parvum*, detected (*n*, %)	7 (18.9)	12 (19.4)	0.950
*Ureaplasma urealyticum*, detected (*n*, %)	7 (18.9)	4 (6.5)	0.090
Delivery time (gestational weeks)	35.71 (32.72–36.57)	39.43 (38.14–40.00)	<0.001
Mode of delivery			0.038
Spontaneous vaginal delivery (*n*, %)	15 (40.54%)	39 (61.90%)	
Cesarean delivery (*n*, %)	22 (59.46%)	24 (38.10%)	
Birth weight (g)	2605.00 (1820.00–2895.00)	3200.00 (2850.00–3425.00)	<0.001

Continuous variables are presented as median (interquartile range, IQR) and compared between groups using the Mann–Whitney U test. Categorical variables are presented as number (percentage) and compared using the chi-square test. Pathogen detection rates for the five pre-specified key organisms were compared between groups using two-sided Fisher’s exact tests.

### 3.2 Vaginal microbiome composition in sPTB vs. term

#### 3.2.1 Alpha and beta diversity

**Figure 1:**
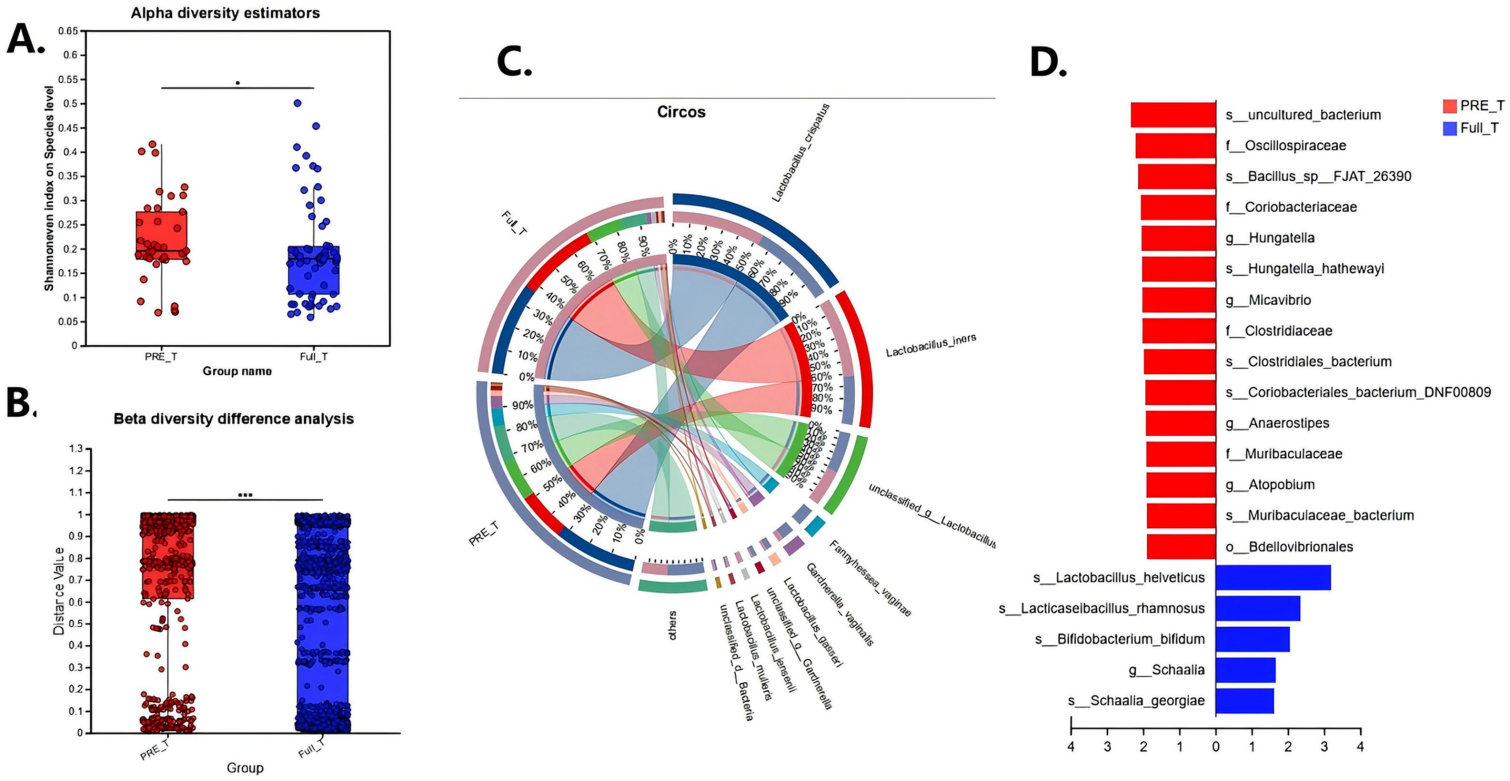
Vaginal microbiome diversity and taxonomic differences in spontaneous preterm birth (sPTB) and term controls. **(A)** Alpha diversity (Shannon index) was significantly higher in sPTB compared with term controls (Wilcoxon rank-sum test). **(B)** Beta diversity (Bray–Curtis distances) also differed significantly between groups (PERMANOVA). **(C)** Circos diagram showing the relative contributions of dominant taxa in the two groups. **(D)** Differentially abundant taxa identified between sPTB (red) and term (blue) groups, highlighting enrichment of BV-associated genera in sPTB and protective taxa (*Lactobacillus helveticus*, *L. rhamnosus*, *Bifidobacterium bifidum*) in term deliveries.

The sPTB and term groups differed markedly in vaginal microbiota diversity. Alpha diversity was higher in the sPTB group (median Shannon index 0.21 vs. 0.19 in term, *p* < 0.05; Figure 1A), indicating that preterm-delivered women had a broader range of vaginal taxa. Most term samples were dominated by a single *Lactobacillus* species, whereas sPTB samples tended to have mixed bacterial communities. For beta diversity, Bray–Curtis dissimilarity-based PCoA demonstrated that overall community composition differed between groups, with sPTB samples clustering apart from term controls (Figure 1B). This between-sample separation indicates a shift in vaginal community structure in sPTB beyond within-sample diversity changes. Consistently, PERMANOVA (999 permutations) confirmed that group status explained a significant proportion of the variation in community structure even after adjustment for potential covariates such as parity (*p* < 0.001; Figure 1C). In concert, the findings are evocative of the switch from the typically low-diversity, *Lactobacillus*-rich state of healthy term pregnancy to a higher diversity, dysbiotic state in sPTB women, in accordance with directions in non-Asian populations (Feehily et al., 2020; Fettweis et al., 2019; Freitas et al., 2018).

#### 3.2.2 Taxonomic differences

Distinct differences in taxonomic composition were also observed. Differential abundance analysis in Figure 1D revealed group-specific microbial patterns. Notably, although women with symptomatic vaginitis at sampling were excluded, several organisms commonly considered pathogens (including *Chlamydia trachomatis*) were still detected in a subset of participants, consistent with asymptomatic carriage; therefore, group ‘enrichment’ in differential-abundance testing should be interpreted alongside between-group detection rates (Table 1) rather than only relative abundance among carriers. In the sPTB group, some of the taxa that are typically observed in relation to inflammation or dysbiosis were enriched, i.e., *Hungatella hathewayi*, *Clostridiales bacterium*, *Coriobacteriaceae*, and *Micavibrio*. Notably, *Bdellovibrionales*, which is an infrequent predatory taxon, was enriched, which signifies the acquisition of environmentally or non-native microbiota among preterm individuals. These observations are consistent with the established ssociation between bacterial vaginosis–like microbiota and preterm birth (Schuster et al., 2024).

On the other hand, group was abundant in helpful taxa like *Lactobacillus helveticus*, *L. rhamnosus*, and *Bifidobacterium bifidum*. They are lactic acid-producers, vaginal pH is low, and inhibit pathogen growth.

Overall, relative abundance of *Lactobacillus* was significantly lower in sPTB cases (mean 73.5% vs. 90.4% in term controls, *p* < 0.05), in keeping with the reduced *Lactobacillus* predominance and resultant risk of preterm birth described previously (Feehily et al., 2020; Fettweis et al., 2019). Consistent with this, sPTB microbiomes had greater relative abundances of several non-*Lactobacillus* species that are archetypal BV-associated anaerobes optimally adapted to grow under high-pH, lactobacillus-deficient conditions.

### 3.3 Metabolomic profiling reveals distinct signatures in sPTB

#### 3.3.1 Overview of study cohorts and sample profiling

**Figure 2:**
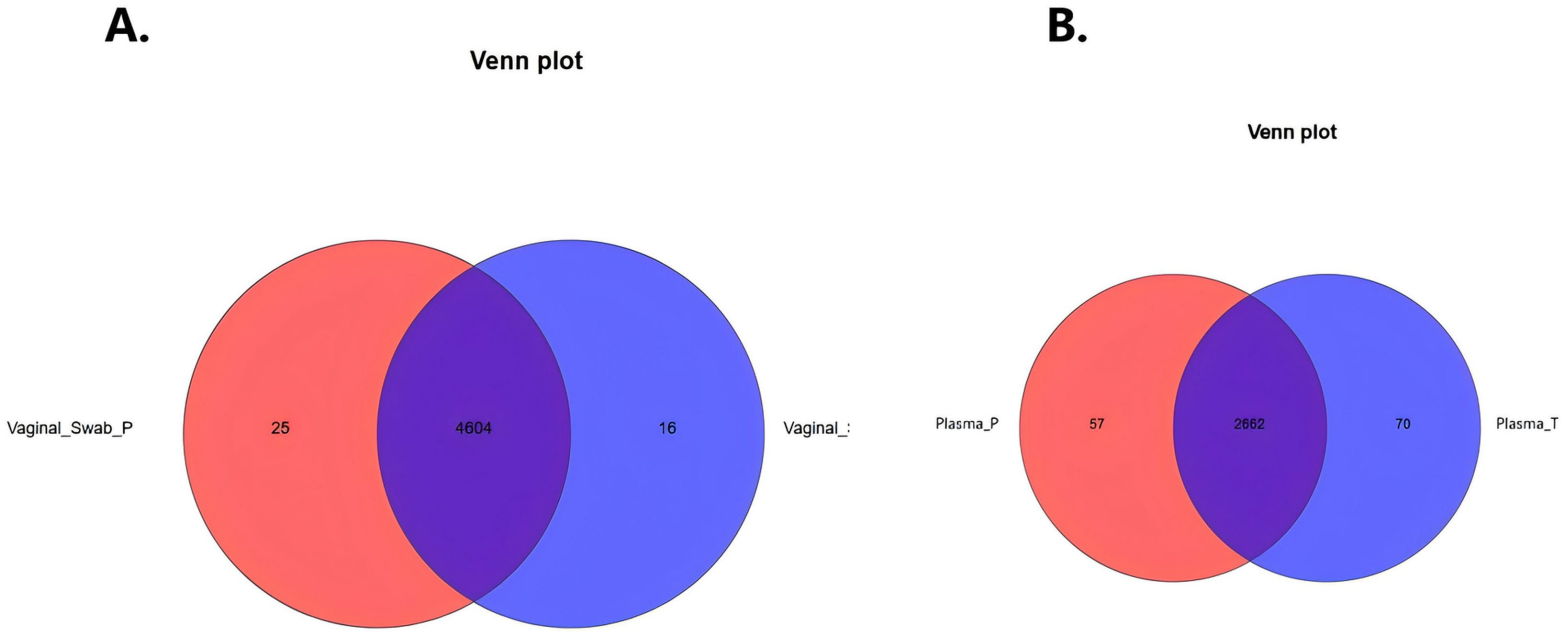
Venn diagrams of metabolite features detected in spontaneous preterm birth (sPTB) and term groups. **(A)** Vaginal swab metabolomic features: 25 features unique to sPTB, 16 unique to term, and 4,604 shared. **(B)** Plasma metabolomic features: 57 features unique to sPTB, 70 unique to term, and 2,662 shared.

Plasma and vaginal swab samples were collected from two groups of Chinese pregnant women: the preterm birth group (Plasma_P and Vaginal_Swab_P) and the full-term birth group (Plasma_T and Vaginal_Swab_T). Untargeted metabolomic profiling was conducted under both cationic and anionic ionization modes. Venn diagram analysis revealed that the majority of metabolites found were shared between groups, yet each group also had their unique characteristics. Specifically, when vaginal swabs were compared, both shared and group-specific metabolites were represented (Figure 2A), while in plasma samples, there were more Plasma_P-specific metabolites, consistent with broader systemic metabolic remodeling associated with sPTB in this cohort (Figure 2B).

#### 3.3.2 Principal component analysis (PCA) and clustering

**Figure 3:**
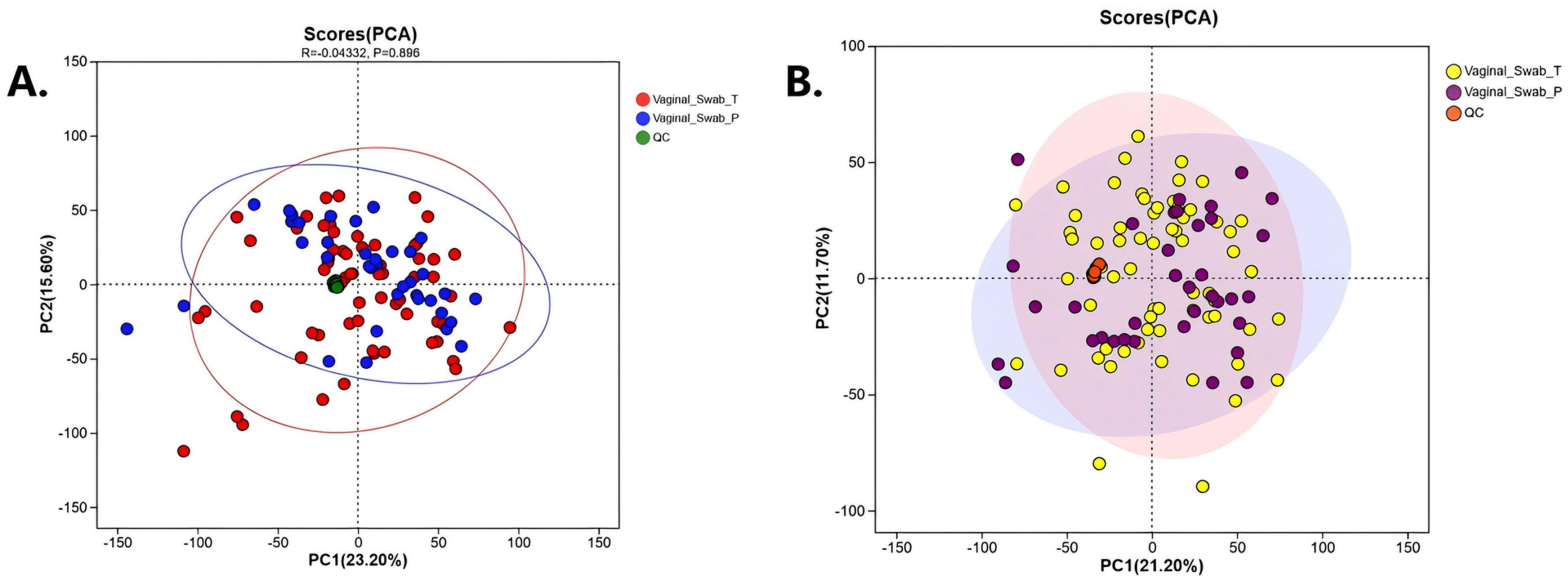
Principal component analysis (PCA) of vaginal metabolomic profiles in spontaneous preterm birth (sPTB) and term controls. **(A)** PCA score plot of vaginal swab samples (sPTB vs. term) showing partial clustering of groups. **(B)** PCA score plot of vaginal swab samples in a separate ionization mode, with quality control (QC) samples clustering tightly in the center, indicating analytical stability.

PCA in an unsupervised manner clearly showed strong spatial discrimination among Plasma_P and Plasma_T plasma samples with both the ion modes (Figures 3A,B). In contrast, vaginal-fluid/swab metabolomes showed weaker global separation on PCA, but the higher within-group dispersion in sPTB suggested a consistent shift toward a more heterogeneous, dysregulated local metabolic state that aligns with sPTB status.

#### 3.3.3 Supervised discriminant analysis (PLS-DA)

**Figure 4:**
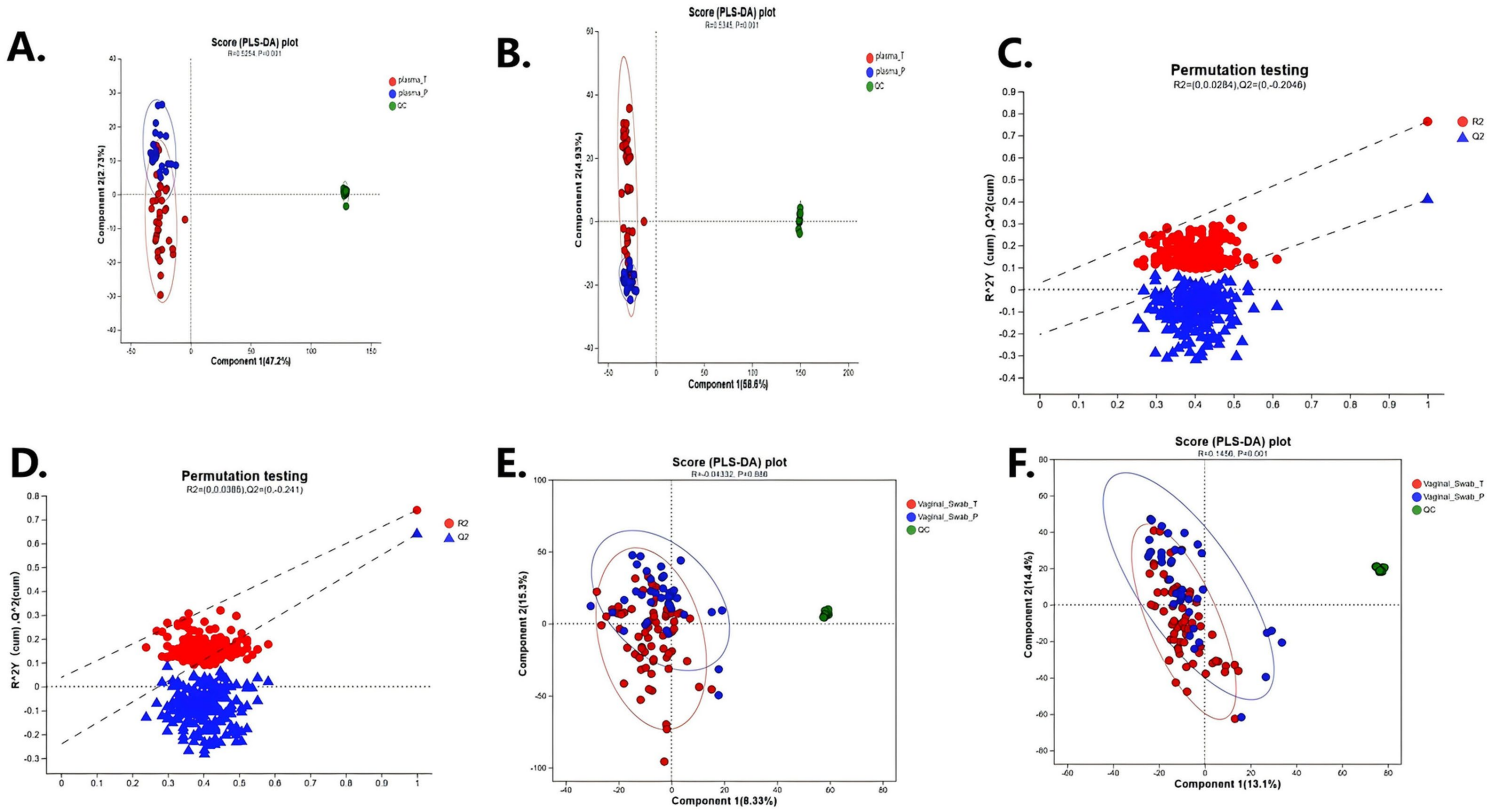
Partial least squares discriminant analysis (PLS-DA) of plasma and vaginal metabolomes in spontaneous preterm birth (sPTB) and term controls. **(A–C)** Plasma metabolome in cationic mode. **(A,B)** PLS-DA score plots showing clear separation between sPTB (Plasma_P) and term (Plasma_T) groups, with QC samples clustering tightly. **(C)** Permutation testing confirming model validity. **(D–F)** Vaginal metabolome in anionic mode. **(E,F)** PLS-DA score plots showing partial separation of sPTB (Vaginal_Swab_P) and term (Vaginal_Swab_T) groups, with QC samples clustering tightly. **(D)** Permutation testing supporting model stability.

PLS-DA modeling confirmed the group-level separation identified by PCA. Plasma PLS-DA models (both cationic and anionic) demonstrated excellent classification performance with high Q^2^ and R^2^ values (Figures 4A,B), and their robustness was confirmed by permutation testing (Figures 4C,D). Vaginal swab models showed more modest but statistically significant group separation (Figures 4E,F), indicating subtle yet consistent local metabolic alterations.

#### 3.3.4 Differential metabolite screening

**Figure 5:**
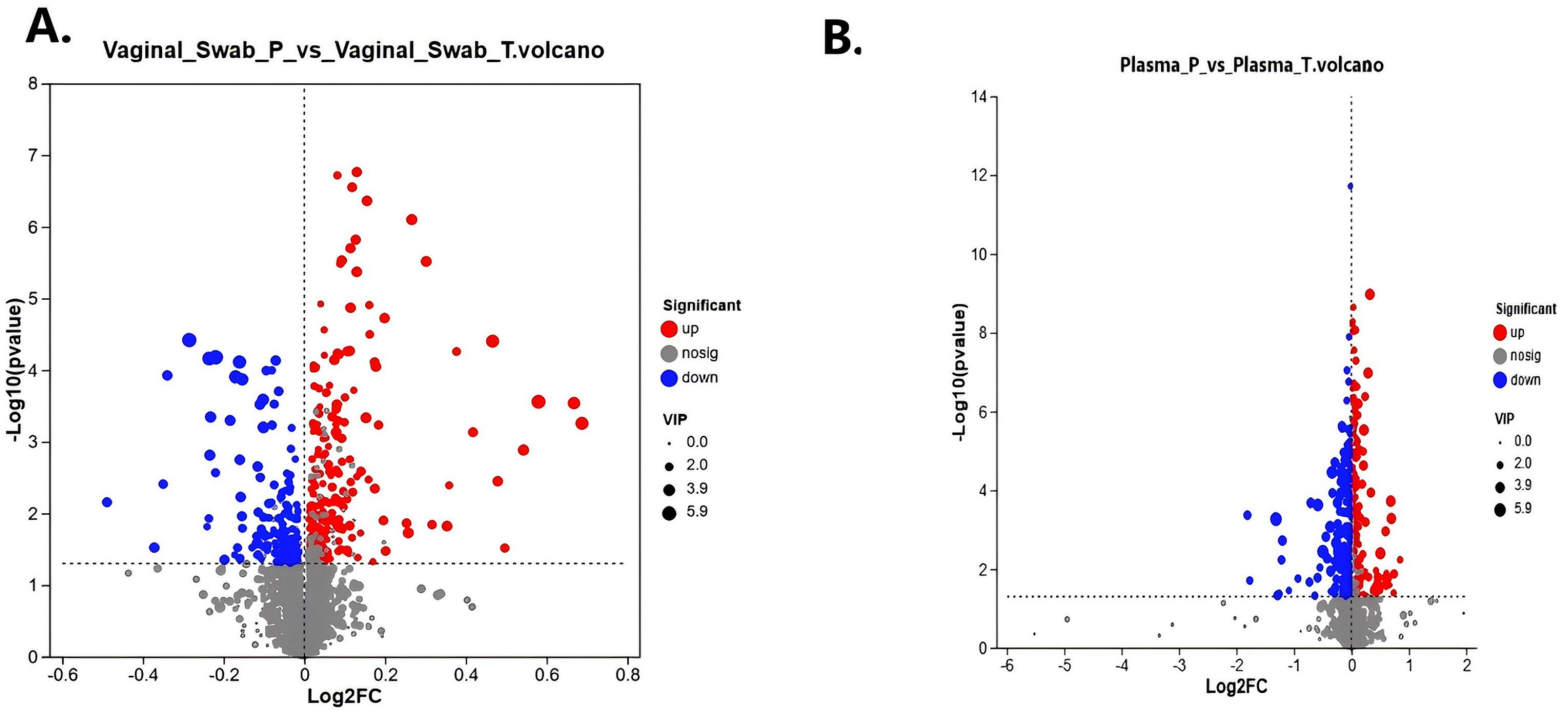
Volcano plots of differential metabolites in vaginal swabs and plasma between spontaneous preterm birth (sPTB) and term groups. **(A)** Vaginal swab metabolites. **(B)** Plasma metabolites. Each point represents one metabolite; red = upregulated in sPTB, blue = downregulated, gray = not significant. The *x*-axis shows log_2_ fold change, and the *y*-axis shows –log_10_(*p* value). Dot size corresponds to variable importance in projection (VIP) score. Differential metabolites were defined as VIP > 1 and false discovery rate (FDR)–adjusted *q* < 0.05, as described in Methods.

Volcano plots (Figure 5) identified several significantly altered metabolites between Plasma_P and Plasma_T in both matrices. Plasma samples yielded a larger number of differential metabolites (|log₂FC| > 1, *p* < 0.05), consistent with stronger systemic alterations. Venn analysis identified metabolites that were differentially abundant in both vaginal and plasma compartments (Supplementary Figure 1), revealing 48 overlapping features; this overlap is descriptive and does not, by itself, imply vaginal-to-systemic causality.

#### 3.3.5 Pathway enrichment analysis

As an exploratory cross-compartment comparison, the 48 overlapping differential metabolites were subjected to KEGG enrichment analysis to highlight pathways concurrently perturbed in both compartments, including vitamin B6 metabolism, pantothenate and CoA biosynthesis, autophagy, glycosylphosphatidylinositol (GPI)-anchor biosynthesis, and *Escherichia coli* infection pathways (Supplementary Figure 2). These findings indicate dysregulation of nutrient metabolism, stress response pathways, and immune-related processes associated with sPTB, consistent with prior studies linking systemic micronutrient status and metabolic perturbations to preterm birth risk (Hughes et al., 2022; Johns et al., 2017). The enrichment of autophagy-related pathways aligns with previous research implicating cellular stress response mechanisms in spontaneous preterm labor (Vora et al., 2020), without implying a specific vaginal-to-systemic causal pathway. Importantly, the presence of a metabolite in both vaginal fluid and plasma does not indicate that vaginal microbiota (or local vaginal processes) determine circulating levels.

#### 3.3.6 Individual metabolite patterns

Boxplots and barplots (Supplementary Figures 3, 4) represented expression profiles for 12 representative metabolites, including pantothenic acid, 4-pyridoxic acid, PE(16:0/22:1), riboflavin, and 3-hydroxyoctanoic acid. Most of them exhibited consistent down- or up-regulation in Plasma_P compared with Plasma_T, supporting group-level trends and suggesting potential candidates for biomarkers.

#### 3.3.7 Biomarker evaluation

ROC curve analysis of the selected metabolites demonstrated strong discriminative power. Pantothenic acid (AUC = 0.82; 95% CI, 0.5498–1), 4-pyridoxic acid (AUC = 0.67; 95% CI, 0.3785–0.95), and PE(16:0/22:1) (AUC = 0.52; 95% CI, 0.4857–0.98) showed good sensitivity and specificity for distinguishing Plasma_P from Plasma_T (Supplementary Figure 5). Other metabolites, such as dimethylallyl diphosphate (DMAPP) and 3-hydroxyoctanoic acid, showed moderate diagnostic performance.

#### 3.3.8 Feature prioritization and integrative biomarker selection

**Figure 6:**
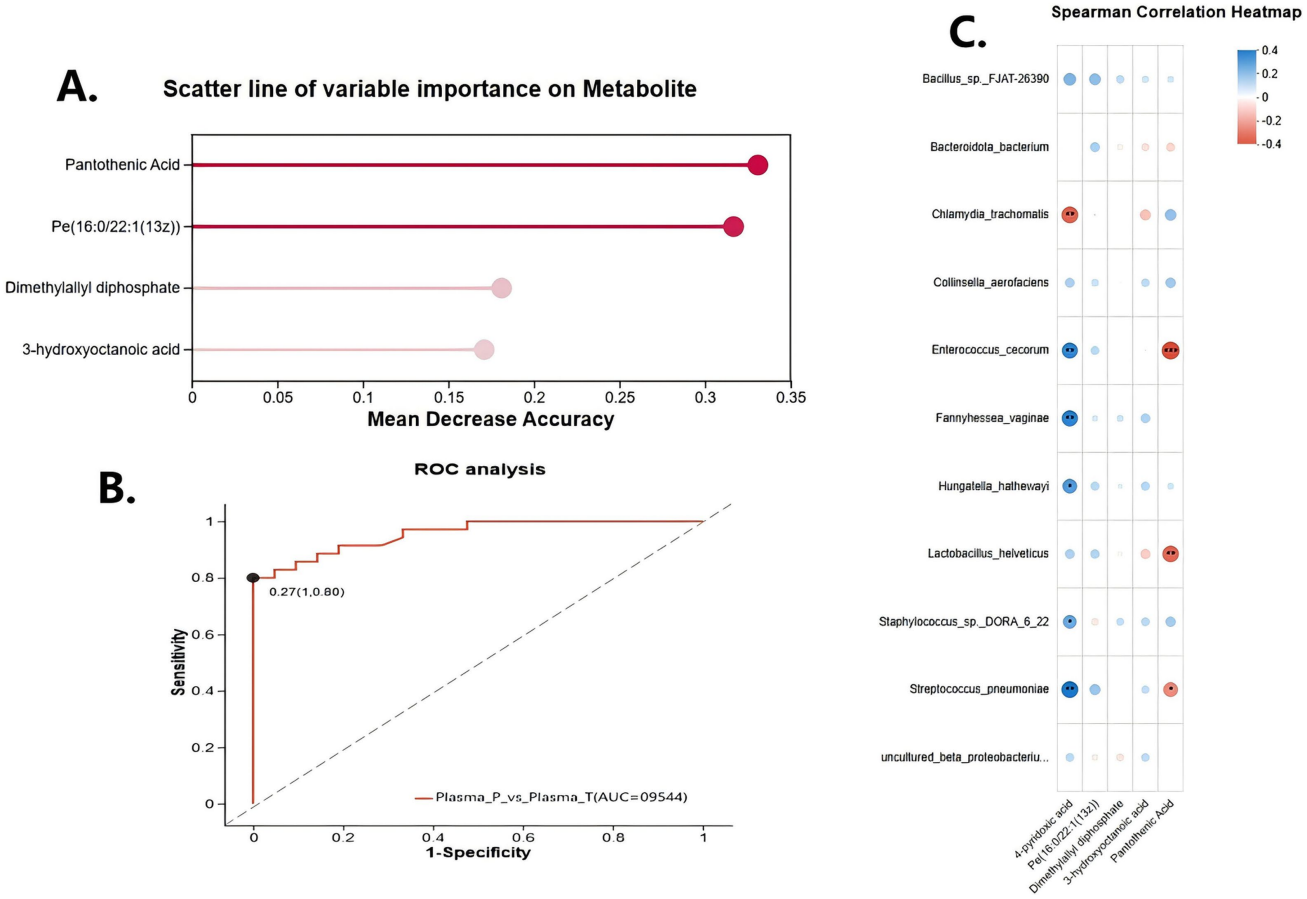
Key metabolite predictors and exploratory microbiome–metabolome correlations in spontaneous preterm birth (sPTB). **(A)** Variable-importance plot from random forest analysis showing the top metabolites contributing to group discrimination (pantothenic acid, PE(16:0/22:1), dimethylallyl diphosphate, and 3-hydroxyoctanoic acid). **(B)** Receiver operating characteristic (ROC) curve of a metabolite-based classifier distinguishing plasma samples from sPTB (Plasma_P) and term (Plasma_T) groups, with area under the curve (AUC) and 95% confidence interval shown. AUC values and confidence intervals were calculated using ROC analysis with bootstrap resampling (1,000 iterations). **(C)** Exploratory Spearman correlation correlation heatmap illustrating associations between relative abundances of differential vaginal bacterial taxa (rows) and concentrations of significantly altered metabolites (columns) in paired samples. Red = positive correlation; blue = negative correlation; circle size reflects correlation strength.

Random forest modeling ranked pantothenic acid, PE(16:0/22:1), and 4-pyridoxic acid among the top contributors to classification accuracy (Figure 6A). Combined scatter plots of AUC and feature importance (Figure 6B) further supported these metabolites as high-utility biomarkers.

#### 3.3.9 Microbiota–metabolite correlations

Spearman correlation heatmaps (Figure 6C) demonstrated coordinated associations between differential metabolites and vaginal microbiota, highlighting integrated patterns of microbe–metabolite variation rather than isolated changes. Of interest, pantothenic acid and 4-pyridoxic acid were positively associated with *Lactobacillus crispatus* and inversely associated with *Fannyhessea vaginae* and *Gardnerella vaginalis*, suggesting potential crosstalk between host metabolic state and microbiota composition in preterm birth. Taken together, these intersectional findings exploratorily support the hypothesis that the vaginal microbiome and maternal metabolome are functionally networked in sPTB pathogenesis (Cavanagh et al., 2025), although the observed associations may be influenced by unmeasured confounders.

Overall, the metabolomic analysis revealed distinct systemic and local biochemical signatures in Chinese women with sPTB compared to those delivering at term. Dysregulated pathways and candidate metabolites—particularly those involved in vitamin metabolism and host–microbe interaction—showed strong discriminatory capacity and biological significance and are therefore appealing candidates for biomarker discovery and mechanistic investigation.

## 4 Discussion

Herein, we conducted a systematic multi-omics investigation of spontaneous preterm birth (sPTB) in Chinese women and identified co-occurring patterns of vaginal microbial dysbiosis and host metabolic derangement. By harmonizing two previously distinct areas of research—the vaginal microbiome and maternal metabolism—our article submits a conceptual model enabling one to see the overall contribution of the two to sPTB. Our findings indicate that local (vaginal) and systemic (plasma) alterations can co-exist in sPTB, although this observational study cannot determine whether one compartment drives changes in the other. Accordingly, subsequent interpretation focuses on microbiome composition and relative abundance patterns rather than binary pathogen detection.

### 4.1 Vaginal dysbiosis as a trigger for sPTB

Consistent with the extensive body of earlier literature, our study shows that sPTB is strongly associated with disruption of the typical *Lactobacillus*-dominated vaginal microbiome (Feehily et al., 2020; Fettweis et al., 2019; Romero et al., 2014). Among term births, vaginal microbiota was typically dominated by *Lactobacillus helveticus* or *L. iners* that are acid producers have low vaginal pH, and repress the overgrowth of pathogens (Ahrodia et al., 2022; Feehily et al., 2020; Petrova et al., 2013; Ng et al., 2021). In contrast, the majority of sPTB women did not possess high counts of Lactobacillus but had polymicrobial flora with an abundance of BV-associated taxa such as *Gardnerella*, *Sneathia*, and *Prevotella*.

These findings are replicated in European and North American research, wherein high-diversity CST IV populations have been consistently linked with risk of PTB (Apilánez Urquiola et al., 2018; Feehily et al., 2020; Fettweis et al., 2019). Fettweis et al. (2019), for example, observed widespread loss of *Lactobacillus crispatus* in preterm delivery with overenrichment of BVAB1, *Prevotella*, and *Sneathia*—results replicated in the present cohort (Fettweis et al., 2019). Our results hence extend these correlations to an Asian (Chinese) population, showing that the correlation of *Lactobacillus* depletion with sPTB crosses ethnicity despite heterogeneity at baseline population level of microbiota (Ng et al., 2021).

Interestingly, one large Chinese cohort study (Ng et al., 2021) identified no link between mid-pregnancy microbiota and PTB. Rather than contradicting that report, our findings likely reflect differences in sampling windows that capture distinct phases of the preterm birth process: mid-pregnancy profiles may relate to baseline susceptibility, whereas samples obtained closer to delivery may reflect microbial and metabolic states proximal to (or downstream of) processes leading to sPTB. Because our design is cross-sectional and near delivery, it is not suited to resolve when dysbiosis emerges or to support causal inference about timing. Longitudinal, repeated sampling across gestation will be needed to determine whether microbiome shifts in Chinese women precede sPTB earlier in pregnancy, arise later, or evolve dynamically over time.

### 4.2 Host metabolic perturbations in sPTB

One of the more interesting results of our study is the implication of systemic metabolic alterations in women who deliver preterm. We found that sPTB is linked to an atypical metabolic profile in which there is decreased pantothenic acid (B5), impaired vitamin B6 catabolism reflected by increased 4-pyridoxic acid, and lipid and amino acid metabolite derangements. Vitamin B6 (pyridoxine) is particularly interesting. It also plays a role in neurotransmitter synthesis, hormone regulation, and immune function – all of which are related to pregnancy maintenance. Vitamin B6 deficiency has also been associated with increased inflammation. Our result of ~2.85% greater 4-pyridoxic acid (the major B6 catabolite) in vaginal fluid in the sPTB group than in term controls suggests that mothers may have had altered B6 metabolism, potentially reflecting increased usage under stress. A pilot first-trimester micronutrient survey also found that low maternal B6 levels were independently associated with PTB risk (Barinov et al., 2022). Taken together, these findings suggest that vitamin B6 status and/or metabolism may differ in women with sPTB; however, because dietary intake and supplement use were not assessed in this cohort, we cannot determine whether the observed differences reflect true inadequacy, altered metabolism, or dietary variation between groups. Randomized trials from the 1980s indicated that pyridoxine supplementation reduced the incidence of preeclampsia and, in some cases, preterm labor (Salam et al., 2015), although these findings were not widely adopted. Our data provide mechanistic plausibility: B6 is a cofactor for enzymes in tryptophan-kynurenine metabolism; deficiency could exacerbate the production of toxic metabolites or weaken collagen cross-linking in fetal membranes. It may be worth revisiting pyridoxine supplementation in a targeted manner (e.g., in women with low levels or high risk of PTB).

Pantothenic acid (vitamin B5) is utilized in coenzyme A biosynthesis and energy metabolism. Deficiency of pantothenate is rare (once so named from “pantothen,” everywhere), but marginal deficiency can result from inadequate diet. In animal studies, B5 deficiency has been shown to reduce adrenal function and increase susceptibility to infection (Tian et al., 2021). In our study, pantothenic acid levels were consistently lower in sPTB women than in term controls, consistent with systemic depletion. The reduction may reflect differences in intake, supplement exposure, absorption/utilization, or disease-related metabolic remodeling; because dietary intake and supplement use were not captured, the relative contribution of nutritional inadequacy versus altered metabolism cannot be disentangled. Interestingly, an additional recent npj examination of the cervicovaginal metabolome (Cavanagh et al., 2025) found higher levels of pantothenate in women who delivered preterm at more progressed gestation, possibly as a result of microbial local production, whereas our plasma data probably reflect host depletion. Pantothenic acid is included in many prenatal supplements; nevertheless, any recommendation for nutrient-specific supplementation in this population should be made cautiously and ideally be guided by direct assessment of dietary intake, supplement use, and biochemical status to confirm inadequacy rather than dietary variation alone. Together, these microbiologic and metabolic results emphasize the multifactorial cause of sPTB and create justification for combination prevention.

### 4.3 Strengths and limitations

One of the greatest strengths of our study is the multi-omics approach incorporated in which we got a complete picture of the pathophysiology of sPTB. We utilized shotgun metagenomics rather than 16S rRNA gene sequencing to facilitate species-level identification (which is crucial for such an infection as *Chlamydia trachomatis*) and to enable the identification of non-bacterial DNA. We also measured both local (vaginal) and systemic (plasma) metabolites, a move few previous studies have made together.

However, there are some limitations to be mentioned. The study was observational and cross-sectional with a single sampling time point in mid-gestation; hence, we cannot establish causality or fully distinguish cause from effect. Although samples were collected prior to delivery and before labor onset/rupture of membranes, the observed microbial and metabolic differences may still reflect early or evolving pathophysiologic processes rather than definitive predictors of sPTB. Longitudinal sampling in early pregnancy will be required to detect predictive alterations. Our cohort size, while moderate, was probably too small to have great statistical power to discern more subtle associations and likely over-estimated some effect estimates; larger cohorts will be required to further drill down these findings. This limitation is most relevant for plasma metabolomics, where only 56 of 99 participants had available plasma (21 sPTB and 35 term), limiting power and potentially yielding unstable estimates given the large number of metabolites tested. Restricting analyses to this subset may introduce selection bias; therefore, characteristics of participants with versus without plasma were compared (Supplementary materials). In addition, the high dimensionality relative to sample size raises concerns about overfitting despite cross-validation, and plasma findings should be considered exploratory and validated in independent cohorts. Restricting our study is also the absence of comprehensive host immune profiling: we had to derive inflammatory status from the metabolites, but simultaneous measurement of cytokines or immune cell activation would much more definitively establish the inflammatory milieu. Also, our group has been enrolled from a single hospital in China, and this may restrict the generalizability of our results. Baseline dietary patterns and vitamin consumption may impact metabolomic profiles; however, we did not collect participant-level data on dietary intake, supplement use, or food security. As a result, we cannot determine whether the observed differences in pantothenic acid and vitamin B6–related metabolites reflect true nutritional inadequacy, differential metabolism, or dietary variation between groups. Nonetheless, some of our findings are consistent with other ethnic populations, suggesting broad applicability.

In conclusion, our integrated study reinforces the view that spontaneous preterm birth is a multifactorial syndrome arising at the intersection of infection, microbiome dysbiosis, and host metabolic disturbance. We provide evidence that a breakdown of maternal–fetal homeostasis—manifested by loss of protective vaginal lactobacilli and deficiencies in essential nutrients—creates a permissive environment for preterm labor. These findings are the basis for the recognition of PTB as greater than an obstetric event but rather as a systemic illness with microbial and biochemical determinants. Ultimately, maximizing pregnancy outcome will likely be necessary to include a hybrid plan that reconciles strategies toward microbial ecology and maternal nutrition/immunity. The integrated perspective enabled by research like ours can be used to guide the design of personalized interventions (tailored on a woman’s microbiota and metabolic profile) and prevention (prior to clinical presentation), looking ahead to reduce the global burden of prematurity.

## 5 Conclusion

Spontaneous preterm birth (sPTB) arises from a convergence of disruptions in the maternal vaginal ecosystem and host metabolic milieu. In this study of Chinese women, we found that sPTB is preceded by depletion of the Lactobacillus-dominated vaginal microbiome and the emergence of dysbiotic, pathogen-enriched communities, together with systemic and local metabolic disturbances including vitamin B5/B6 imbalance, heightened tryptophan catabolism, and altered lipid metabolism. These changes may interact synergistically in the pathophysiology of preterm labor: vaginal dysbiosis could promote local inflammation, while systemic metabolic perturbations (captured in plasma) may reflect broader whole-body physiology that can influence immunity and tissue integrity; however, the direction and mechanisms of any vaginal-to-systemic signaling cannot be inferred from cross-sectional associations alone. Our metagenomics–metabolomics approach enabled the identification of putative biomarkers (e.g., *Lactobacillus* level, pantothenic acid, 4-pyridoxic acid) with potential to improve early sPTB prediction. Of particular interest, unlike fixed obstetric risk factors, many of the pathways we outline are potentially modifiable—suggesting that treatments such as microbiome reconstitution, infection assessment, and nutrient-specific supplementation might interrupt the pathologic cycle to preterm birth. These findings offer new insights into the pathogenesis of sPTB in an Asian population and demonstrate the utility of systems biology for multi-factorial obstetric syndromes. Through an explanation of how microbial and metabolic networks in sPTB become dysregulated, this research provides a foundation for designing novel strategies for prolonging gestation and reducing the global burden of prematurity.

## Data Availability

The datasets presented in this article are not readily available because of the sensitivity of the collected medical data and the need to protect participant privacy. Requests to access the datasets should be directed to the corresponding authors, Zhaodong Liu at Lzd20252025@163.com or Mian Pan at panmian1973@126.com.

## Supplementary material

SUPPLEMENTARY FIGURE 1 Overlap of differential metabolites between vaginal swab and plasma matrices. Venn diagram showing the overlap of significantly altered metabolites identified in vaginal swab (red) and plasma (blue) comparisons between sPTB and term groups. A total of 48 metabolites were shared, while 317 and 299 were unique to vaginal swab and plasma, respectively. The bar chart below summarizes the number of differential metabolites identified in each comparison.

SUPPLEMENTARY FIGURE 2 KEGG pathway enrichment analysis of shared differential metabolites. Bubble plot showing enriched KEGG pathways for the 48 overlapping differential metabolites identified in both plasma and vaginal swab comparisons (from Figure 5). The *x*-axis represents the rich factor, bubble size indicates the number of metabolites mapped to each pathway, and bubble color represents the adjusted P value. Significant pathways included vitamin B6 metabolism, pantothenate and CoA biosynthesis, and autophagy-related signaling.

SUPPLEMENTARY FIGURE 3 Differential metabolites in vaginal secretions between spontaneous preterm birth (sPTB) and term groups. Boxplots of representative metabolites showing altered abundance in sPTB (red) compared with term controls (blue): **(A)** PE(16:0/22:1), **(B)** tyramine, **(C)** 4-pyridoxic acid, **(D)** 3-hydroxyoctanoic acid, **(E)** pantothenic acid, and **(F)** dimethylallyl diphosphate. Statistical significance was assessed using Wilcoxon rank-sum test with FDR correction (Methods). Significance levels: *p* < 0.05 (*), *p* < 0.01 (***).

SUPPLEMENTARY FIGURE 4 Differential metabolites in plasma between spontaneous preterm birth (sPTB) and term groups. Boxplots/barplots of representative metabolites showing altered abundance in sPTB (red) compared with term controls (blue): **(A)** PE(16:0/22:1), **(B)** tyramine, **(C)** 4-pyridoxic acid, **(D)** 3-hydroxyoctanoic acid, **(E)** pantothenic acid, and **(F)** dimethylallyl diphosphate. Statistical significance was assessed using Wilcoxon rank-sum test with FDR correction (Methods). Significance codes: *p* < 0.05 (*), *p* < 0.01 (**), *p* < 0.001 (***).

SUPPLEMENTARY FIGURE 5 Diagnostic performance of top metabolite biomarkers for spontaneous preterm birth (sPTB). Receiver operating characteristic (ROC) curves for selected metabolites distinguishing sPTB from term controls: **(A)** pantothenic acid, **(B)** 3-hydroxyoctanoic acid, **(C)** dimethylallyl diphosphate, **(D)** 4-pyridoxic acid, and **(E)** PE(16:0/22:1). Shaded areas represent 95% confidence intervals. Area under the curve (AUC) values with 95% confidence intervals are shown within each panel. AUC values and confidence intervals were calculated using ROC analysis with bootstrap resampling (1000 iterations).
