# Supplementary material for: Integrated metagenomic and metabolomic profiling of spontaneous preterm birth in Chinese women
Source: Front Microbiol. 2026 Mar 18;17:1729476. doi: 10.3389/fmicb.2026.1729476 (PMC13038873; doi:10.3389/fmicb.2026.1729476)
Supplement: Supplementary file 1 [file Presentation_1.PPTX]

## Slide 1
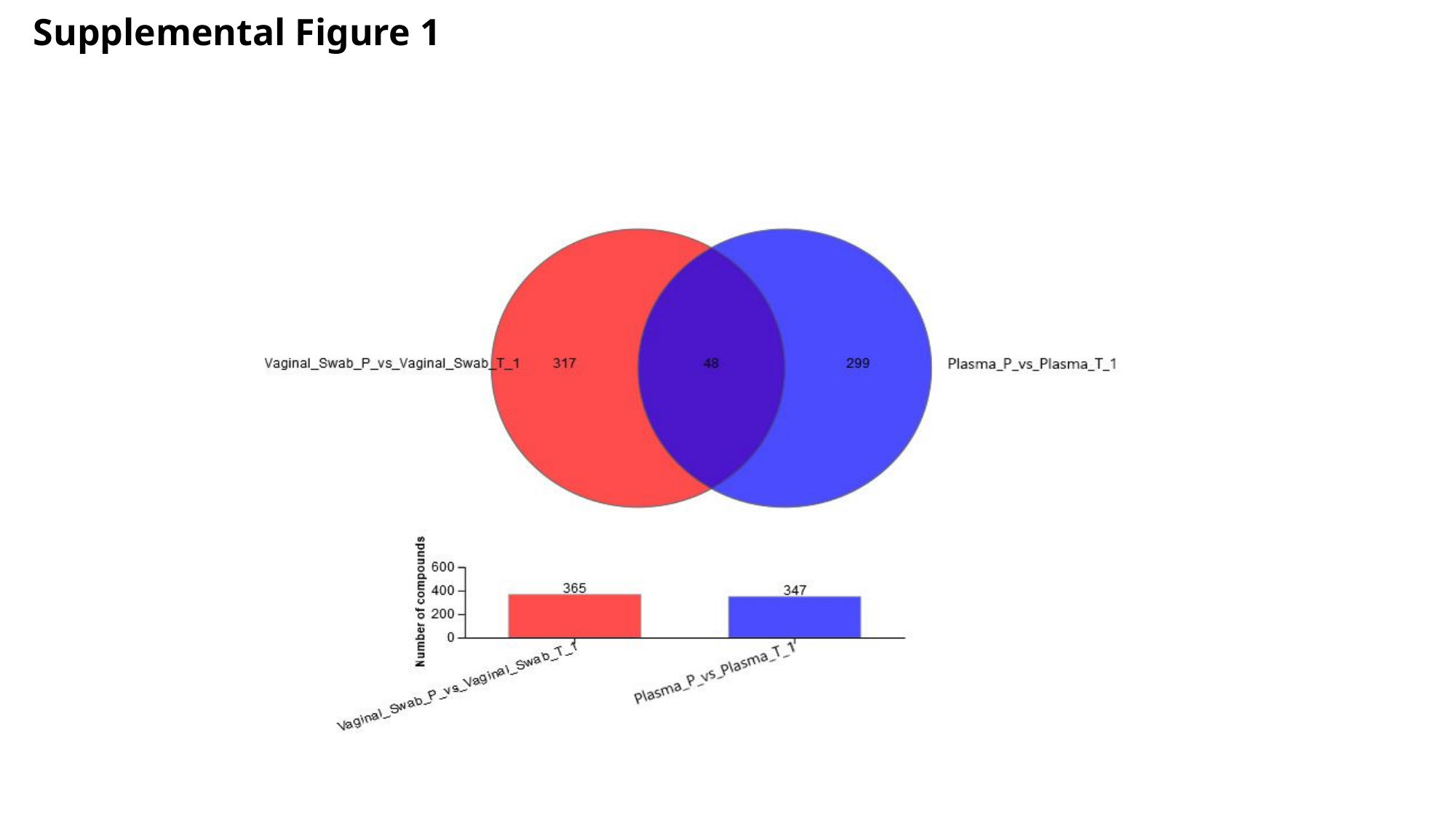

Supplemental Figure 1

## Slide 2
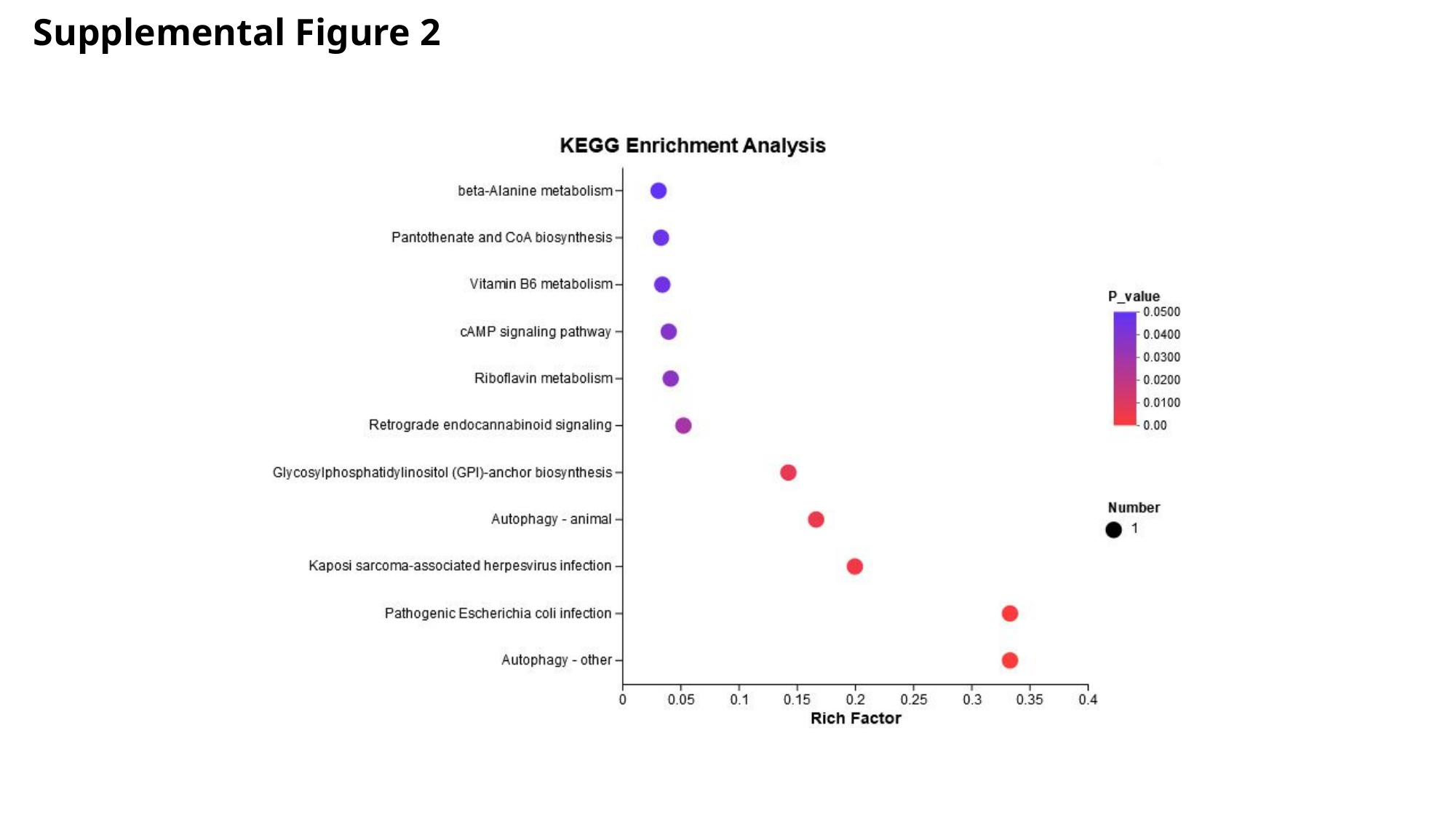

Supplemental Figure 2

## Slide 3
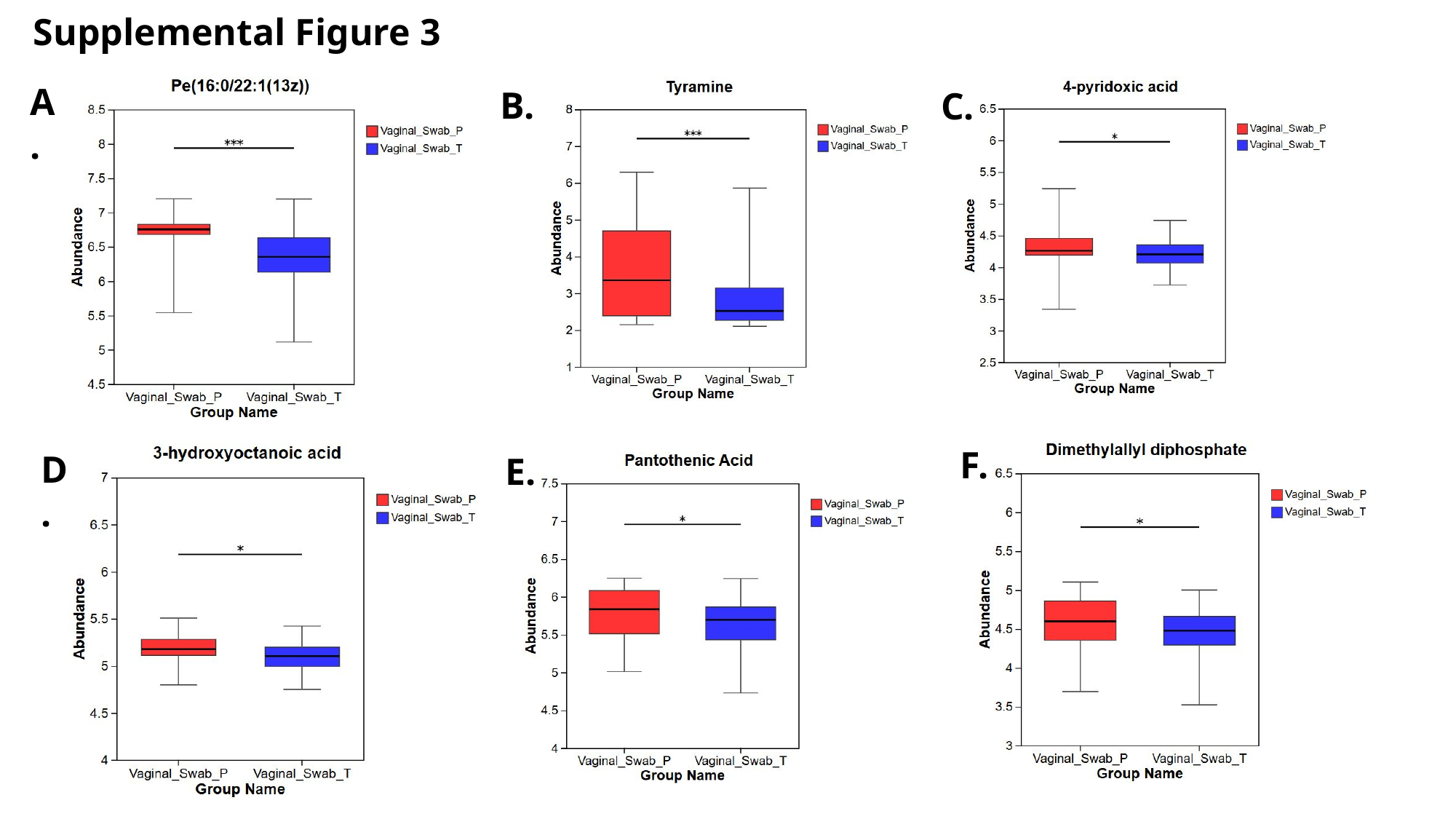

Supplemental Figure 3
A.
B.
C.
F.
D.
E.

## Slide 4
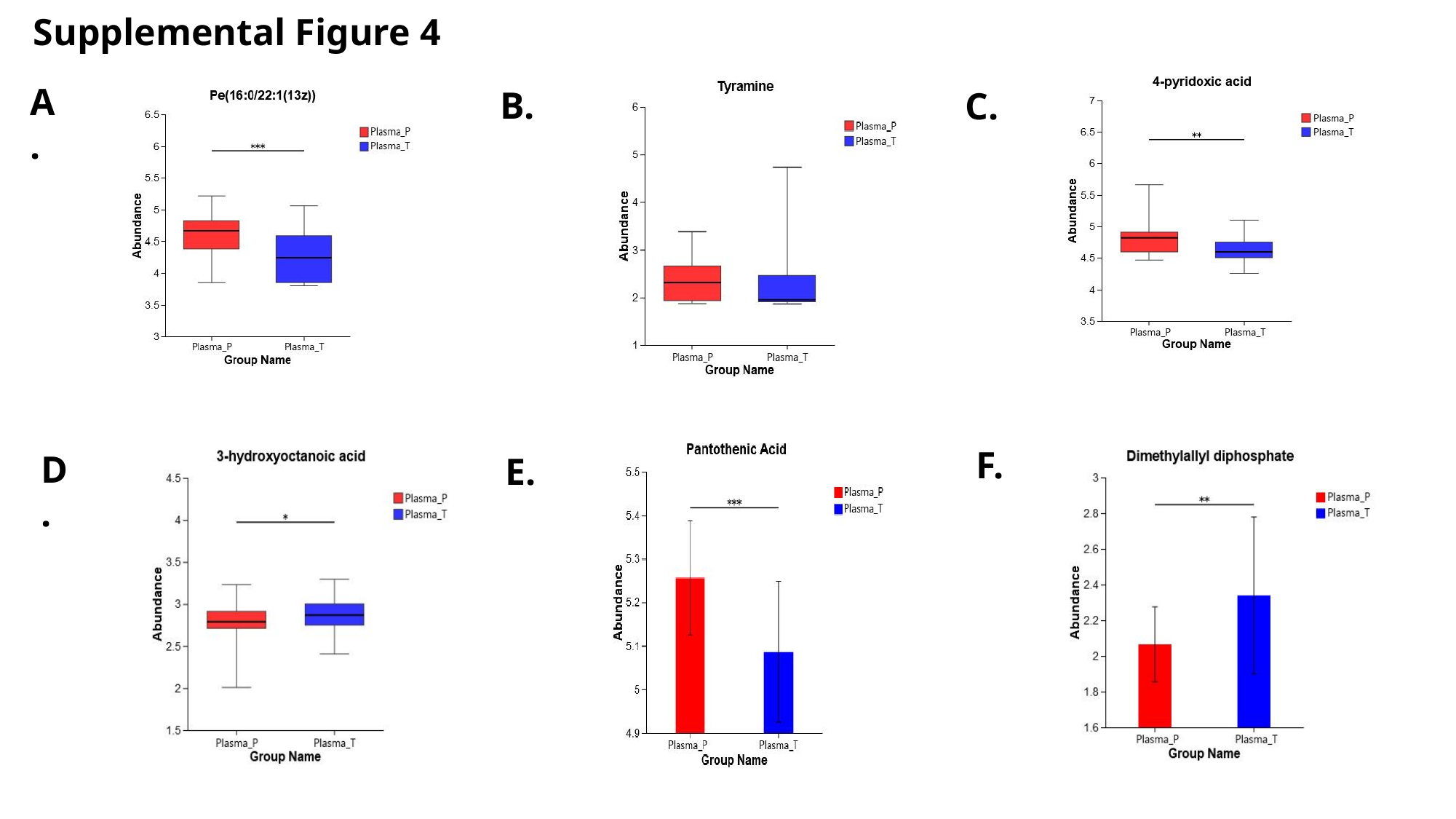

Supplemental Figure 4
A.
B.
C.
F.
D.
E.

## Slide 5
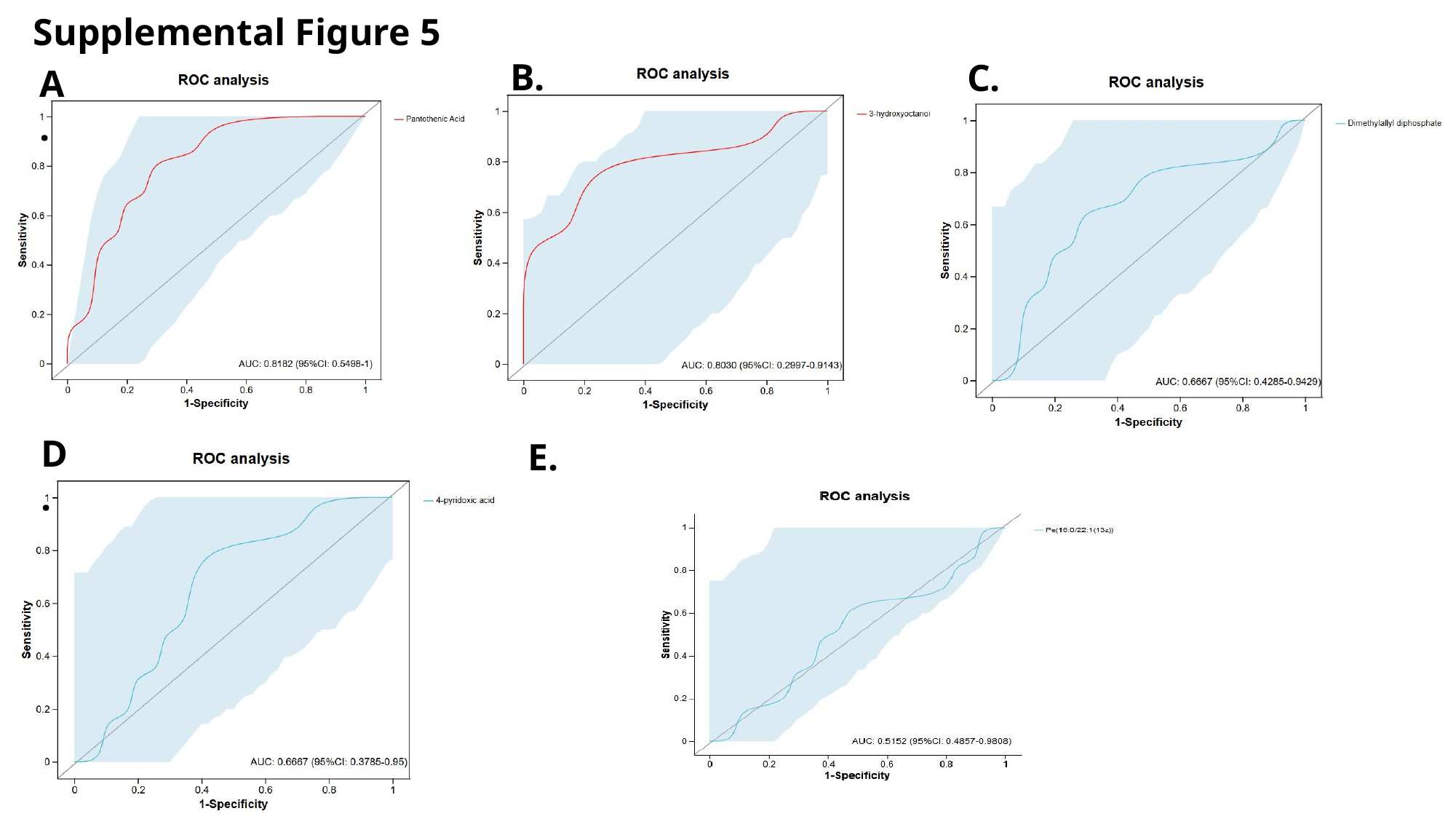

Supplemental Figure 5
B.
C.
A.
D.
E.
